# Pharmacokinetics, Distribution, Metabolism, and Excretion of Omadacycline following a Single Intravenous or Oral Dose of ^14^C-Omadacycline in Rats

**DOI:** 10.1128/AAC.01784-16

**Published:** 2016-12-27

**Authors:** Wen Lin, Jimmy Flarakos, Yancy Du, Wenyu Hu, Handan He, James Mangold, S. Ken Tanaka, Stephen Villano

**Affiliations:** a Novartis Biomedical Research Institute, East Hanover, New Jersey, USA; b Paratek Pharmaceuticals, Boston, Massachusetts, USA

**Keywords:** ADME, aminomethylcycline, animal models, omadacycline

## Abstract

The absorption, distribution, metabolism, and excretion (ADME) of omadacycline, a first-in-class aminomethylcycline antibiotic with a broad spectrum of activity against Gram-positive, Gram-negative, anaerobic, and atypical bacteria, were evaluated in rats. Tissue distribution was investigated by quantitative whole-body autoradiography in male Long-Evans Hooded (LEH) rats. Following an intravenous (i.v.) dose of 5 mg/kg of body weight, radioactivity widely and rapidly distributed into most tissues. The highest tissue-to-blood concentration ratios (t/b) were observed in bone mineral, thyroid gland, and Harderian gland at 24 h post-i.v. dose. There was no evidence of stable accumulation in uveal tract tissue, suggesting the absence of a stable binding interaction with melanin. Following a 90 mg/kg oral dose in LEH rats, the highest t/b were observed in bone mineral, Harderian gland, liver, spleen, and salivary gland. The plasma protein binding levels were 26% in the rat and 15% to 21% in other species. Omadacycline plasma clearance was 1.2 liters/h/kg, and its half-life was 4.6 h; the steady-state volume of distribution (Vss) was 6.89 liters/kg. Major circulating components in plasma were intact omadacycline and its epimer. Consistent with observations in human, approximately 80% of the dose was excreted into the feces as unchanged omadacycline after i.v. administration. Fecal excretion was primarily the result of biliary excretion (∼40%) and direct gastrointestinal secretion (∼30%). However, urinary excretion (∼30%) was equally prominent after i.v. dosing.

## INTRODUCTION

Omadacycline is a first-in-class aminomethylcycline antibiotic with a broad spectrum of microbiologic activity against Gram-positive, Gram-negative, anaerobic, and atypical bacteria. Omadacycline is being developed as a once-daily oral and intravenous (i.v.) therapy for the treatment of acute bacterial skin and skin structure infections (ABSSSI) and community-acquired bacterial pneumonia (CABP). The microbiological activity, pharmacokinetic profile, and potential clinical efficacy and tolerability of omadacycline in preclinical and phase 1 and phase 2 clinical studies have been described previously (1–5).

As part of the drug development process, an understanding of the disposition and metabolism of a novel drug is necessary to fully understand the potential safety implications (6). The safety concern arises when metabolites comprise 10% or more of the systemic exposure of the parent drug. Thus, mass balance studies in laboratory animals using radiolabeled compounds are a standard part of the development process for new drugs (7). Results from mass balance studies in animal models have been reported for most contemporary antibiotics, including dalbavancin, levofloxacin, linezolid, tedizolid, telavancin, and tigecycline (8–14). This study was undertaken to characterize the pharmacokinetics and protein binding as well as the absorption, distribution, metabolism, and excretion (ADME) characteristics of omadacycline following oral and intravenous (i.v.) doses of ^14^C-omadacycline in rat models.

## RESULTS

### Tissue and body fluid distribution.

Following a ^14^C-omadacycline intravenous dose of 5 mg/kg of body weight in rats, concentrations of radioactivity were extensively distributed to tissues. The radioactivity concentration in most tissues was higher than that in blood within 5 min of i.v. dosing and at up to 24 h postdose (Table 1). The radioactivity concentration in bile at 0.083 h postdose was 100-fold higher than that in blood (maximum concentration [*C*_max_], 1,570 ng · eq/ml) but was not measurable at 24 h. The highest tissue-to-blood concentration ratios (t/b) were observed in bone mineral, thyroid gland, and Harderian gland at 24 h postdose, whereas brain, spinal cord, eye, white fat, brown fat, and seminal vesicles showed the lowest tissue-to-blood ratio at both 5 min (t/b < 1) and 24 h (t/b < 3) postdose. The uveal tract had a t/b of 4.8 at 5 min postdose, indicating high distribution into the tissue, but the concentration was undetectable at 24 h, indicating no stable binding interaction with melanin tissue.

**TABLE 1:** Tissue radioactivity concentrations and tissue-to-blood concentration ratios following a 5 mg/kg i.v. dose of ^14^C-omadacycline in LEH rats^*a*^

Tissue	5-min tissue radioactivity concn	t/b	24-h tissue radioactivity concn	t/b
Adrenal cortex	11,300	7.2	323	5.6
Adrenal medulla	5,910	3.8	NM	NA
Bile	157,000	100	NM	NA
Blood	1,570	1.0	57.6	1.0
Bone marrow	6,740	4.3	811	14
Bone mineral	956	0.61	7,490	130
Brain	51.3	0.03	61.1	1.1
Brown fat	7,960	5.1	117	2.0
Colon wall	10,600	6.8	298	5.2
Epididymis	2,490	1.6	98.4	1.7
Esophagus	4,660	3.0	NM	NA
Eye	17.4	0.01	22.9	0.40
Harderian gland	4,290	2.7	1,560	27.1
Heart	9,020	5.7	66.8	1.2
Kidney cortex	12,700	8.1	318	5.5
Kidney medulla	11,500	7.4	167	2.9
Kidney pelvis	12,300	7.9	161	2.8
Liver	11,300	7.2	451	7.8
Lung	3,720	2.4	120	2.1
Lymph node	3,510	2.2	666	11.6
Muscle	3,650	2.3	55.6	1.0
Pancreas	6,970	4.4	103	1.8
Pituitary gland	8,570	5.5	538	9.3
Salivary gland	12,000	7.6	262	4.6
Seminal vesicle(s)	75.5	0.05	166	2.9
Skin	4,420	2.8	378	6.6
Small intestine wall	8,930	5.7	229	4.0
Spinal cord	60.6	0.04	64.4	1.1
Spleen	8,540	5.4	699	12.1
Stomach glandular	7,680	4.9	348	6.0
Testis	579	0.37	413	7.2
Thymus	3,360	2.1	204	3.6
Thyroid gland	7,250	4.6	3,950	68.6
Uveal tract	7,490	4.8	NM	NA
White fat	3.93	0.003	7.83	0.14

a t/b, tissue-to-blood concentration ratios; NM, not measurable because tissue or body fluid was not discernible in the autoradiogram.

Following a single oral 90 mg/kg dose of ^14^C-omadacycline administered to LEH rats, peak tissue concentrations were generally observed at 1 to 7 h postdose in most tissues, with measurable radioactivity. The radioactivity concentration in measurable tissues was higher than in blood (*C*_max_ = 125 ng · eq/ml), except in brain, spinal cord, and eyes (Table 2 and Table 3). At 24 h postdose, the radioactivity in blood and about two-thirds of tissues was below the lower limit of quantification (LLOQ), whereas a substantial amount of radioactivity was measured in bone mineral. The highest tissue-to-blood concentration ratios (t/b > 5), calculated as values corresponding to the area under the concentration-time curve (AUC), were observed in bone mineral, Harderian gland, liver, spleen, and salivary gland (Table 3). Radioactivity exposure with a t/b of 1 to 5 was observed in bone marrow, kidney (cortex, pelvis, and medulla), thymus, heart, adrenal cortex, lung, thyroid gland, and pancreas (Table 3).

**TABLE 2:** Tissue radioactivity concentrations following a 90 mg/kg oral dose of ^14^C-omadacycline in LEH rats^*a*^

Tissue	Tissue radioactivity concn (ng · eq/ml)				
	0.5 h	1 h	3 h	7 h	24 h
Adrenal cortex	NM	1,760	521	354	NM
Adrenal medulla	NM	NM	NM	NM	NM
Bile	NM	NM	NM	NM	NM
Blood	97.9	99.5	125	123	43.6^*b*^
Bone marrow	257	325	474	492	295
Bone mineral	NM	45.3	1,460	1,140	1,380
Brain	41.4	40.3^*b*^	47.1^*b*^	67.3	39.9
Brown fat	NM	NM	NM	NM	NM
Colon wall	NM	239	1,690	3,060	NM
Epididymis	NM	NM	NM	NM	NM
Esophagus	NM	13,600	19,400	4,770	NM
Eye	53.8	NS	74.1^*b*^	40.3	16.0^*b*^
Harderian gland	NM	243	686	1,074	404
Heart	349	330	288	253	108
Kidney, cortex	541	721	627	481	133
Kidney, medulla	532	722	588	402	152
Kidney, pelvis	1,580	832	NS	437	98.7
Liver	1,510	1,100	979	838	292
Lung	287	202	328	179	115
Lymph node	NM	NM	NM	NM	NM
Muscle	115	151	267	142	37.0^*b*^
Pancreas	NM	479	463	608	NM
Pituitary gland	NM	346	219	154	NM
Salivary gland	708	746	884	974	104
Seminal vesicles	NM	NM	NM	NM	NM
Skin	NM	NM	330	395	NM
Small intestine wall	NM	NM	7,031	2,150	NM
Spinal cord	8.65^*b*^	50.6	43.3^*b*^	77.3	51.7^*b*^
Spleen	1,490	1,680	1,140	555	196
Stomach, glandular	NM	5,940	1,527	NM	359
Testis	89.4	49.6	171	101	76.2^*b*^
Thymus	179	164	322	NM	186
Thyroid gland	NM	411	605	729	NM
Uveal tract	NM	NM	NM	NM	NM
White fat	NM	23.5^*b*^	8.89^*b*^	NM	45.2^*b*^

a NM, not measurable because tissue or body fluid was not discernible in the autoradiogram; NS, not sampled.

b Values below the lower limit of quantitation, not included in the PK calculation.

**TABLE 3:** Tissue pharmacokinetic parameters following a single oral dose of 90 mg/kg ^14^C-omadacycline in LEH rats^*a*^

Tissue	*T*_max_ (h)	*C*_max_ (ng · eq/g)	AUC_last_ (ng · eq · h/g)	AUC_0–∞_ (ng · eq · h/g)	*t*_1/2_ (h)	t/b	
						*C*_max_	AUC_last_
Blood	3	125	2,210	3,030	13	1.0	1.0
Adrenal cortex	1	1,760	4,470	5,920	2.9	14.1	2.02
Adrenal medulla	NA	NA	NA	NA	NA	NA	NA
Bile	NA	NA	NA	NA	NA	NA	NA
Bone marrow	7	492	9,630	NA	NA	3.94	4.36
Bone mineral	3	1,460	28,100	NA	NA	11.7	12.7
Brain	7	67.3	364	NA	NA	0.53	0.16
Brown fat	NA	NA	NA	NA	NA	NA	NA
Colon wall	7	3,060	11,500	NA	NA	24.5	5.20
Epididymis	NA	NA	NA	NA	NA	NA	NA
Esophagus	3	19,400	84,800	109,000	3.5	155	38.4
Eye	3	74.1	173	NA	NA	0.59	0.078
Harderian gland	7	1,070	17,100	NA	NA	8.56	7.74
Heart	0.5	349	5,030	7,320	15	2.79	2.28
Kidney cortex	1	721	9,240	11,000	9.3	5.77	4.18
Kidney medulla	1	722	8,450	10,900	11	5.78	3.82
Kidney pelvis	0.5	1,580	9,360	10,500	7.9	12.6	4.24
Liver	0.5	1,510	16,400	21,300	12	12.1	7.42
Lung	3	328	4,240	6,900	16	2.62	1.92
Lymph node	NA	NA	NA	NA	NA	NA	NA
Muscle	3	267	1,330	NA	NA	2.14	0.60
Pancreas	7	608	3,200	NA	NA	4.86	1.45
Pituitary gland	1	346	1,400	2,590	5.4	2.77	0.63
Salivary gland	7	974	15,000	NA	NA	7.79	6.79
Seminal vesicle(s)	NA	NA	NA	NA	NA	NA	NA
Skin	7	395	1,860	NA	NA	3.16	0.84
Small intestine wall	3	7,030	27,100	NA	NA	56.2	12.3
Spinal cord	7	77.3	409	NA	NA	0.62	0.19
Spleen	1	1,680	13,800	16,300	9	13.4	6.24
Stomach, glandular	1	5,940	28,800	32,300	6.9	47.5	13.0
Testis	3	171	822	NA	NA	1.37	0.37
Thymus	3	322	5,960	NA	NA	2.58	2.70
Thyroid gland	7	729	3,790	NA	NA	5.83	1.71
Uveal tract	NA	NA	NA	NA	NA	NA	NA
White fat	NA	NA	NA	NA	NA	NA	NA

a Tissue-to-blood concentration ratios (t/b) were calculated using either AUC_last_ or *C*_max_. NA: not applicable.

Drug-related radioactivity after a 90 mg/kg oral dose of ^14^C-omadacycline showed a low distribution to the central nervous system (brain and spinal cord), since the concentrations were low (<80 ng · eq/g) and <LLOQ for most time points, although the tissue-to-blood concentration ratios were 0.62 (based on *C*_max_) and ∼0.19 (based on AUC). The tissue-to-blood concentration ratios of 1.37 (based on *C*_max_) and 0.37 (based on AUC) suggested moderate distribution of drug-related radioactivity to the testis (Table 3).

### *In vitro* protein binding.

Omadacycline was weakly bound to plasma proteins of all tested species, with no major species differences. In the omadacycline concentration range of 10 to 10,000 ng/ml, no obvious concentration dependency of plasma protein binding was found. The mean unbound protein fractions in plasma were 84.7% ± 5.3% in mouse, 73.9% ± 12.1% in rat, 78.8% ± 7.3% in monkey, and 78.7% ± 9.7% in human.

### Pharmacokinetics, metabolism, and excretion of omadacycline.

**FIG 1:**
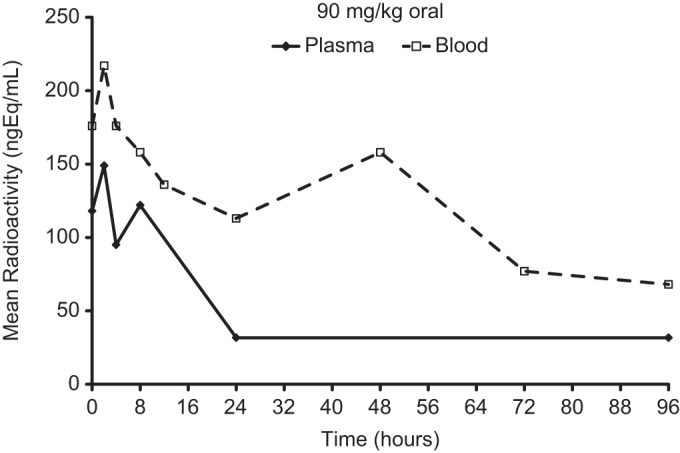
Mean concentrations of total radioactivity (quantified as nanogram equivalents per milliliter) in blood and plasma following a 90 mg/kg oral dose of ^14^C-omadacycline to Han/Wistar (HW) rats (*n* = 3).

**FIG 2:**
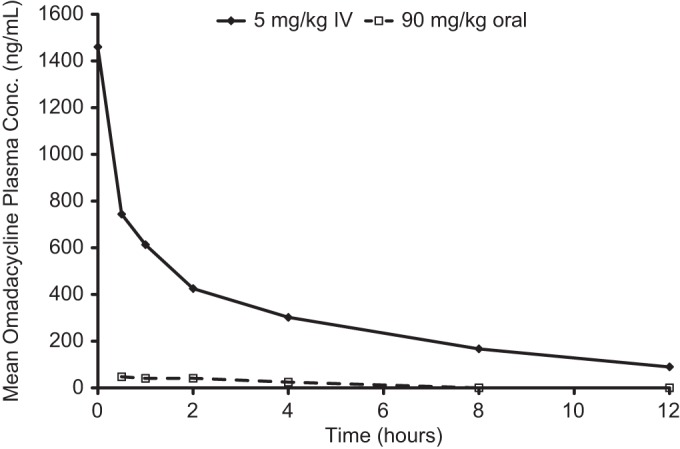
Mean concentrations of omadacycline in rat plasma following a single i.v. dose of 5 mg/kg ^14^C-omadacycline or an oral dose of 90 mg/kg ^14^C-omadacycline to HW rats (*n* = 3).

Following a 90 mg/kg oral dose of ^14^C-omadacycline, the peak radioactivity concentration in plasma (*C*_max_, 172 ng · eq/ml) was attained at between 0.25 and 2 h (Fig. 1). The peak plasma concentration of unchanged omadacycline (*C*_max_, 47.5 ng/ml) was attained at 0.5 h after oral dosing (Fig. 2), further suggesting rapid absorption (Table 4). Absorption of omadacycline was estimated to be 2.9% based on the oral-to-i.v. ratio of the values corresponding to the dose-normalized area under the concentration/time curve at last observation (AUC_last_) of radioactivity in plasma. The oral bioavailability was very low (0.23%) compared to the absorption (Table 4), suggesting significant first-pass elimination. The oral bioavailability was much lower than ∼35% in humans (5).

**TABLE 4:** Mean pharmacokinetic parameters of omadacycline in rat plasma after a single 5 mg/kg i.v. dose or 90 mg/kg oral dose of ^14^C-omadacycline

Parameter^*a*^	^14^C-Omadacycline value	
	5 mg/kg i.v. dose (*n* = 3)	90 mg/kg oral dose (*n* = 3)
*t*_1/2_ (h)	4.6	3.9
*T*_max_ (h)	0.083	0.5
*C*_max_ (ng/ml)	1,460.0	47.5
AUC_last_ (ng · h/ml)	3,590.0	146.0
AUC_0–∞/dose_ ([ng · h/ml]/[mg/kg])	836	3.1
AUC_0–∞_ (ng · h/ml)	4,180.0	283.0
CL (ml/h/kg)	1,200.0	
*V*_ss_ (ml/kg)	6,890.0	
Absorption (%)		2.93
Bioavailability (%)		0.226

a AUC_last_, area under the concentration/time curve at last observation; AUC_0–∞/dose_, area under the concentration/time curve from 0 h to infinity adjusted for dose; AUC_0–∞_, area under the concentration/time curve from 0 h to infinity; CL, clearance; *C*_max_, peak concentration of drug in plasma; *T*_max_, time to peak concentration of drug in plasma; *t*_1/2_, half-life; *V*_ss_, volume of distribution (steady state).

**FIG 3:**
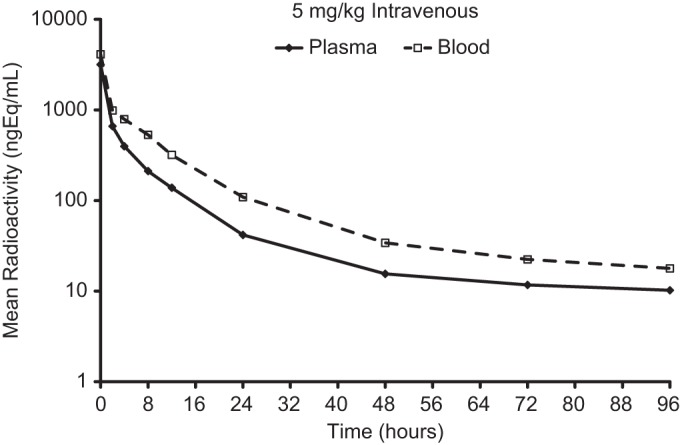
Mean concentrations of total radioactivity in blood and plasma following a single 5 mg/kg i.v. dose of ^14^C-omadacycline to HW rats (*n* = 3).

Following a 5 mg/kg i.v. dose of ^14^C-omadacycline, the mean total radioactivity concentration (4,110 ng · eq/ml) at 5 min postdose in blood rapidly declined to ∼18% at 4 h and was ∼3% at 24 h. At 4 h, plasma concentrations of ^14^C-omadacycline-related radioactivity declined rapidly to ∼12% of the initial concentration (3,170 ng · eq/ml) (Fig. 3). Systemic plasma clearance (CL; 1,200 ml/h/kg) appeared to be moderate compared to the level in the hepatic blood flow in the rat (3.3 liters/h/kg), assuming equal distributions of ^14^C-omadacycline in the blood and plasma. The plasma volume of distribution at the steady state of the unchanged compound (*V*_ss_; 6.89 liters/kg) was larger than the body water volume (0.6 liters/kg), suggesting that omadacycline was extensively distributed to tissues.

The extraction recovery of ^14^C-omadacycline and its metabolites in intact rat plasma was approximately 70% (i.v. dose only). The predominant radioactive components in rat plasma after a single i.v. dose were unchanged (omadacycline/C-4 epimer, 89.9% AUC). The level of M30 metabolite represented 2.6% of the AUC, and impurity-2 represented 6.3% AUC. Due to low levels of detected radioactivity in intact rat, the oral dose in plasma was not analyzed.

After the i.v. dose, the majority of radioactivity (73.4% to 85.8% mean, 80.4%) was excreted in the feces (Table 5). Following i.v. administration, approximately 30% of the dose was recovered in bile, ∼35% in feces, and ∼40% in urine (Table 6). Equal levels of excretion from bile and direct gastrointestinal secretion yielded about 70% to 80% fecal excretion of omadacycline in rats. Urine excretion accounted for about 28% to 38% of the dose in intact or bile duct-cannulated (BDC) rats (Table 5 and Table 6). Omadacycline/C-4 epimer together were unchanged and were the major components in bile, urine, and feces, accounting for 27.3%, 39.7%, and 30.2% of the i.v. dose and for 95.9% of the i.v. dose. Thus, the metabolism of omadacycline/C-4 epimer was limited, and omadacycline/C-4 epimer together were primarily eliminated unchanged in rats. Several minor metabolites were derived from N-demethylation and mono-oxygenation. The results suggested that omadacycline was mainly eliminated via excretion and not via metabolism in rats.

**TABLE 5:** Excretion of parent omadacycline and total radioactivity in the intact HW rats^*a*^

Route of excretion, time of excretion	Amt excreted (% of dose)			
	i.v. (5 mg/kg)		Oral (90 mg/kg)	
	Radioactivity	Omadacycline	Radioactivity	Omadacycline
Feces, 0–24 h	61.0 ± 18.1	56.7	117 ± 2.0	
Feces, 0–168 h	80.4 ± 6.3		120 ± 3.9	84.4
Urine, 0–24 h	25.1 ± 2.20	24.9	0.237 ± 0.016	ND
Urine, 0–48h	30.0 ± 1.49		0.289 ± 0.011	
Total amt recovered	112 ± 5.5		120 ± 3.9	

a All data are means ± standard deviations or means. ND, not detected.

**TABLE 6:** Excretion of parent omadacycline and total radioactivity in the BDC rats^*a*^

Route of excretion, time of excretion	Amt excreted (% of dose)	
	Radioactivity	Omadacycline
Bile, 0–24 h	28.6 ± 2.55	27.3
Bile, 0–48 h	29.6 ± 2.47	
Feces, 0–24 h	27.1 ± 9.93	
Feces, 0–168 h	35.4 ± 5.08	30.2
Urine, 0–24 h	39.1 ± 6.33	
Urine, 0–48 h	42.5 ± 7.28	38.4
Total amt recovered	109 ± 3.96	

a All data are means ± standard deviations or means. ND, not detected.

After the oral dose, most (∼120%) radioactivity was recovered in feces, primarily due to unabsorbed material. Only trace radioactivity (0.29% of the dose) was detected in the urine sample due to poor absorption. The mass balance recovered in excreta within 168 h postdose was complete for both dose groups.

**FIG 4:**
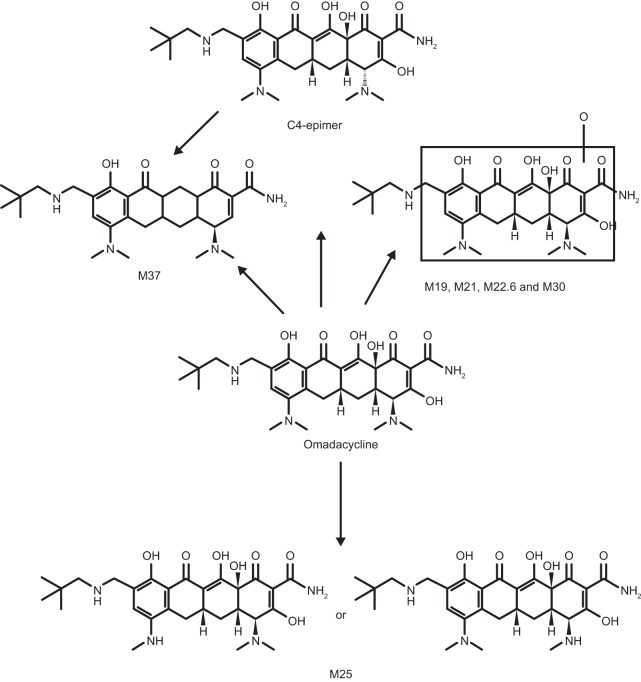
Proposed metabolic scheme of ^14^C-omadacycline in the HW rats.

The mean radioactivity in urine accounted for ∼30% of the i.v. dose and 0.29% of the oral dose. The major radioactivity peaks were those of omadacycline and the C-4 epimer, representing 20.6% and 4.3% of the i.v. dose. Three minor metabolites (M25, M30, and M37) each accounted for <1% the i.v. dose (Fig. 4). Because only 0.29% of the oral dose was recovered from intact rat urine, these data were not analyzed. Unchanged omadacycline and its C-4 epimer were the major components of intact rat feces at 56.7% and 84.4% of the i.v. and oral dose, respectively. Metabolites M25 and M37 were detected at 15.7% and 1.7% of the i.v. dose, respectively. The M37 metabolite also was detected in the feces at 7.0% of the oral dose.

## DISCUSSION

Results from this study showed that absorption of omadacycline in the rat was rapid and bioavailability was low. The poor absorption and bioavailability may be the result of bile salt interaction with omadacycline in the small intestine; omadacycline achieved high concentrations in bile and small intestine after i.v. administration and high concentrations in small intestine after oral administration. In humans, bioavailability was estimated to be approximately 35% (5). In contrast to humans and monkeys, rats lack gallbladder organs; therefore, bile flow in rats is continuous. In the presence of a relatively higher concentration of bile salt in rat small intestine, omadacycline may interact with bile salt and be entrapped in bile salt micelles (15). Consequently, the absorption may be greatly reduced in rats. Omadacycline displayed moderate clearance and a high *V*_ss_ level in rats which were consistent with the pharmacokinetics (PK) parameters in healthy volunteers after i.v. administration (5,500 ml/kg) (16). After a 300-mg oral dose of omadacycline was administered to healthy subjects, the maximum concentration of drug in plasma (*C*_max_) was 0.5 μg/ml, the time to maximum concentration of drug in plasma (*T*_max_) was 3.0 h, the half-life was 16.8 h, and the area under the concentration-time curve from 0 h to infinity (AUC_0–∞_) was 10.3 μg · h/ml, which are substantially greater than the values seen after oral administration of omadacycline in the present study (4).

Omadacycline was widely distributed into tissues and bile in rats, with tissue-to-blood ratios exceeding 1 in most tissues. The highest tissue-to-blood ratios were observed in bone, Harderian gland, liver, spleen, and salivary gland. Of note, tissue levels exceeded blood levels in lung, kidney, skin, bone, and epididymis and/or testis after both oral and i.v. single-dose administration. The tissue-to-blood ratio of >2 in lung is of particular relevance given the clinical development of omadacycline for the treatment of CABP. Further, because these assessments were based on blood radioactivity concentrations, which were approximately 1.3-fold higher than those seen in plasma at 5 min after i.v. administration (Fig. 4), and because omadacycline concentrations are measured in plasma in humans, it would be expected that the human tissue-to-plasma concentration ratio for omadacycline would be higher. The lack of stable binding to uveal tract tissue suggests no stable interaction with melanin tissue.

Excretion of omadacycline, consisting of equal levels of biliary excretion and direct gastrointestinal secretion, was predominantly seen in the feces, although approximately 30% of systemic omadacycline was eliminated in the urine. A primary involvement of P-glycoprotein in omadacycline transport across Caco-2 monolayers was observed, which suggests that biliary excretion may occur via P-glycoprotein *in vivo*. Parent omadacycline and its C-4 epimer, which represents a tetracycline impurity (17), were the primary components in plasma following both oral and i.v. administration. These results indicate that elimination of omadacycline occurs via excretion rather than metabolism in rats. Two inactive metabolites, M25 and M37, were recovered, but these represented 15% or less of the administered dose of omadacycline. No measurable levels of metabolites of omadacycline have been identified in humans to date, an absence which contributes to a low risk of drug-drug interactions.

In contrast to omadacycline, tigecycline undergoes extensive metabolism to eight metabolites in humans such that parent tigecycline represents only 27% of total excretion over a 48-hour period (18). The M5 and M6 metabolites of tigecycline demonstrated antimicrobial activity but at a lower level than the parent compound. The impact of extensive metabolism on antimicrobial activity, pharmacokinetics, and safety and tolerability may be difficult to predict.

Mass balance studies performed with other antibiotics have identified limited tissue distribution of dalbavancin, linezolid, and tedizolid (8, 9, 12, 19), more prominent urinary elimination of delafloxacin, linezolid, and telavancin (9, 10, 20), and the presence of metabolites of delafloxacin, solithromycin, and telavancin (10, 20, 21). All these factors may impact on the efficacy, safety, and tolerability profile of any antibiotic.

In summary, in rats, omadacycline is characterized by high biliary, fecal, and renal excretion, low absorption, low lipophilicity (clog *P* = <1), and high aqueous solubility. Omadacycline distributes to tissues associated with common infectious diseases such as pneumonia and infections of the skin and urinary tract. In addition, plasma protein binding was <30%, which may have been beneficial because the free, unbound fraction of an antibiotic typically is most closely correlated with antimicrobial activity. The limited metabolism in rats is encouraging in regard to human metabolism and drug interactions. These results suggest the potential use of omadacycline in the treatment of a variety of human infections caused by susceptible bacterial pathogens.

## MATERIALS AND METHODS

### Chemicals.

**FIG 5:**
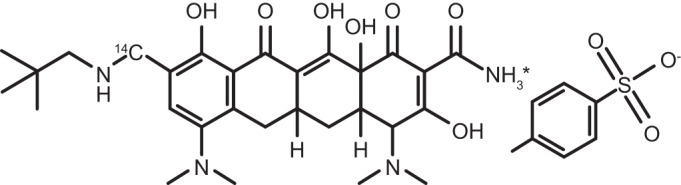
Chemical structures of ^14^C-omadacycline and the internal standard (C292H6H34N4O7).

^14^C-Omadacycline was synthesized by the Isotope Laboratory of Novartis Pharmaceuticals Corporation (East Hanover, NJ, USA). The specific activities were 20 μCi/mg (i.v.) and 1.65 μCi/mg (oral). The purity of the compound was >97%. The internal standard for the analysis of unchanged omadacycline was [^13^C_6_], provided by Paratek Pharmaceuticals (Boston, MA, USA). The chemical structure of radiolabeled omadacycline and position of the radiolabel are shown in Fig. 5.

### Tissue and body fluid distribution via QWBA.

Distribution of radioactivity of omadacycline into tissues, organs, and body fluids was investigated by quantitative whole-body autoradiography (QWBA). Pigmented Long-Evans Hooded (LEH) rats were administered a single oral dose (*n* = 8) of ^14^C-omadacycline at 90 mg/kg (1.92 μCi/mg) via gavage and a single i.v. dose (*n* = 2) of ^14^C-omadacycline at 5 mg/kg (20 μCi/mg) with a 30-min infusion via a jugular catheter. Animals were sacrificed 0.5, 1, 3, 7, 24, 48, and 168 h following a single oral dose and 0.083 and 24 h following a single i.v. dose.

Following sacrifice, animal carcasses were frozen and then sectioned in specimens of 40-μm thickness. Sections analyzed included adrenal gland (cortex and medulla), artery, bile, blood, bone (marrow and mineral), brain, colon wall, epididymis, esophagus, eye, fat (brown and white), Harderian gland, heart, kidney (cortex, medulla, and pelvis), lachrymal gland, liver, lung, lymph node, muscle, pancreas, pituitary gland, salivary gland, seminal vesicles, skin, small intestine wall, spinal cord, spleen, stomach (nonglandular and glandular linings), testis, thymus, thyroid gland, and uveal tract. A block of ^14^C-radiolabeled standards, prepared in blood and assayed by liquid scintillation counting, was sectioned in the same manner and on the same days as each rat was sectioned.

To assess the actual concentrations in calibration standards and quality control samples, replicate 50-μl aliquots of spiked blood were analyzed for total radioactivity. The values (expressed in numbers of disintegrations per minute [DPM] per milliliter) obtained from the radio assay of the spiked blood samples and digital analysis of the spots on the section blocks (molecular dynamic count [MDC] per square millimeter × 10^3^) were used to generate the calibration curve used to calculate the actual ^14^C concentrations in tissues, organs, and body fluids. The levels of radioactivity present in tissues were determined by digital analysis of the resulting phosphor image autoradiogram. The tissue sections were placed in a light-tight cassette in contact with a storage phosphor screen for exposure and shielded in a lead box. After 72 h (i.v.) or 168 h (oral), the autoradiogram of tissue sections derived from rats dosed with i.v. drug was developed by scanning the storage phosphor screens using a Molecular Dynamics Storm 860 scanner (i.v.) or GE Storm 865 scanner (oral). Tissue radioactivity concentrations were determined by digital analysis of the resulting autoradiogram.

### Pharmacokinetics, metabolism, and excretion of omadacycline.

The pharmacokinetics of ^14^C-omadacycline were determined in Han/Wistar male rats. The oral group (*n* = 3) received ^14^C-omadacycline at 90 mg/kg (1.65 μCi/mg) via gavage after an 8-h fast, and those in the i.v. group (*n* = 3) received ^14^C-omadacycline at 5 mg/kg (20.0 μCi/mg) by slow bolus injection via a jugular catheter. Approximately 250 μl of blood was collected using an automatic sample collection system (Culex; BASi, West Lafayette, IN). The samples were delivered to preheparinized tubes in 4°C sample collection carousels and stored until further processing. Saline solution (250 μl) was automatically injected after each sample was collected to clear the cannula and replace the volume of blood taken.

Serial blood and plasma samples were collected at 0.083 (i.v. only), 0.25, 0.5, 1, 2, 4, 8, 12, 24, 48, 72, 96, and 168 h after the start of dosing; urine and feces were collected over 0 to 168 h using 24-h intervals; cage wash was collected at 168 h postdose. Radioactivity was analyzed in all plasma, blood, urine, feces, and cage wash samples.

Excretion of omadacycline following an i.v. dose of ^14^C-omadacycline in the bile duct-cannulated (BDC) rats was investigated. BDC rats had two catheters surgically implanted, into both the bile duct and jugular veins. Bile was collected from each rat over periods 0 to 4, 4 to 8, 8 to 12, 12 to 24, and 24 to 48 h postdose under ice-cooling conditions. Urine and feces were collected at 0 to 24 h and 24 to 48 h, and cage wash samples were collected at 48 h postdose.

For analysis of metabolites, pooled (*n* = 3 for i.v. and oral dose groups) plasma and selected pooled urine, bile, and feces samples were analyzed for unchanged omadacycline and metabolites. ^14^C-Omadacycline and its metabolites in the plasma and excreta were analyzed by high-pressure liquid chromatography (HPLC) with offline radioactivity detection. Omadacycline and metabolite concentrations in rat plasma (expressed as nanograms per equivalent per milliliter) were calculated by multiplying the percent peak of a metabolite in the radiochromatogram (expressed as a fraction) by the radioactivity in plasma expressed as nanograms per equivalent per milliliter. Levels of metabolites and parent omadacycline in urine, bile, and feces were quantified as percentages of the dose. No correction for the extraction recovery was included.

Omadacycline and its internal standard were isolated from sodium heparin-pooled rat plasma samples (50 μl) using a 96-well solid-phase extraction procedure. For the initial extraction steps, samples were thawed, subjected to vortex mixing for 1 min, and centrifuged for 10 min at 3,000 rpm. A 500-μl volume of 2% Na_2_EDTA(aq) was added to each well in a 2.4-ml 96-deep-well collection plate. A 25-μl volume of internal standard (ISTD) working solution (5 μg/ml of omadacycline-d6–water) was added to all wells except control blanks. A 25-μl volume of Milli-Q water (prepared in-house) was added to control blank wells. The plate was centrifuged at 3,000 rpm for up to 1 min to ensure that all liquid was in the bottom of the wells. Volumes (50 μl) of standard control (C), quality control (QC), or pooled rat plasma samples were then added to the appropriate wells. The plate was sealed with a cap mat and subjected to vortex mixing to ensure thorough mixing.

An Oasis hydrophilic-lipophilic-balanced (HLB) 96-well extraction plate (Waters, Milford, MA, USA) (10 mg) was conditioned on a Tomtec Quadra system (Hamden, CT, USA) with 400 μl of methanol, followed by conditioning with 400 μl of Milli-Q water. Samples were then transferred to the extraction plate and drawn through using low vacuum. The wells were washed with 400 μl of Milli-Q water, followed by another wash with 400 μl of methanol/water (5:95 [vol/vol]). The plate was placed on high vacuum for approximately 2 min to remove remaining water. The samples were then eluted with 400 μl of methanol into a 1-ml 96-deep-well block.

The eluent was evaporated to dryness under nitrogen at 45°C and reconstituted with 100 μl of Milli-Q water. The reconstituted samples were sealed and subjected to vortex mixing for 1 min and then centrifuged for 5 min at 3,000 rpm. Samples were then transferred to a Corning Costar half-height block (Tewksbury, MA, USA) using a personal pipettor for analysis on the mass spectrometer.

All pharmacokinetic parameters were calculated with the computer program WinNonlin (S3; Certara, Princeton, NJ). The highest average plasma omadacycline and radioactivity concentrations (*C*_max_) and corresponding times (*T*_max_) were recorded. For the i.v. dose, the first sampling time was at 0.083 h. The concentration profiles of the radioactivity of omadacycline in blood and plasma were analyzed using WinNonlin and the pharmacokinetic parameters, including the area under the concentration curve from h 0 to infinity (AUC_0–∞_), and the terminal half-lives were estimated by a noncompartmental analysis. Clearance (CL) and the steady-state volume of distribution (*V*_ss_) of omadacycline were calculated using data from the i.v. dose. The fraction or percentage of the dose absorbed was calculated based on blood or plasma radioactivity data, assuming a proportional relationship between AUC and dose. For the major metabolites in plasma, *C*_max_ and *T*_max_ were recorded as observed. The AUC from h 0 to h 48 (AUC_0–48_) was calculated using the linear trapezoidal rule.

### *In vitro* plasma protein binding.

Plasma protein binding of omadacycline at concentrations of 10, 100, 1,000, and 10,000 ng/ml was evaluated in mouse (CD-1), rat (Han/Wistar), monkey (cynomolgus), and human using defrosted plasma pools. Protein binding was determined by an EMD Millipore Centrifree ultrafiltration method (EMD Millipore, Billerica, MA, USA) following incubation at 37°C for 30 min after spiking using liquid scintillation counting (control test) and ultraperformance liquid chromatography-tandem mass spectrometry (UPLC-MS/MS).

## References

[1] Draper MP, Weir S, Macone A, Donatelli J, Trieber CA, Tanaka SK, Levy SB 2014 Mechanism of action of the novel aminomethylcycline antibiotic omadacycline. Antimicrob Agents Chemother 58:1–11. doi:10.1128/AAC.01066-13. 24041885 PMC3957880

[2] Macone AB, Caruso BK, Leahy RG, Donatelli J, Weir S, Draper MP, Tanaka SK, Levy SB 2014 In vitro and in vivo antibacterial activities of omadacycline, a novel aminomethylcycline. Antimicrob Agents Chemother 58:1127–1135. doi:10.1128/AAC.01242-13. 24295985 PMC3910882

[3] Noel GJ, Draper MP, Hait H, Tanaka SK, Arbeit RD 2012 A randomized, evaluator-blind, phase 2 study comparing the safety and efficacy of omadacycline to those of linezolid for treatment of complicated skin and skin structure infections. Antimicrob Agents Chemother 56:5650–5654. doi:10.1128/AAC.00948-12. 22908151 PMC3486554

[4] Sun H, Ting L, Maietta R, Machineni S, Praestgaard J, Kuemmell A, Stein DS, Sunkara G, Kovacs SJ, Villano S, Tanaka SK 10 2016 A randomized, open-label study of the pharmacokinetics and safety of oral and intravenous administration of omadacycline in healthy subjects. Antimicrob Agents Chemother doi:10.1128/AAC.01393-16. PMC5119026 27736760

[5] Flarakos J, Du Y, Gu H, Wang L, Einolf HJ, Chun DY, Zhu B, Alexander N, Natrillo A, Hanna I, Ting L, Zhou W, Dole K, Sun H, Kovacs SJ, Stein DS, Tanaka SK, Villano S, Mangold JB 8 2016 Disposition, metabolism, and drug-drug interaction properties of omadacycline. Xenobiotica doi:10.1080/00498254.2016.1213465. 27499331

[6] FDA. 2 2008 Guidance for industry, safety testing of drug metabolites. http://www.fda.gov/downloads/Drugs/.../Guidances/ucm079266.pdf.

[7] Roffey SJ, Obach RS, Gedge JI, Smith DA 2007 What is the objective of the mass balance study? A retrospective analysis of data in animal and human excretion studies employing radiolabeled drugs. Drug Metab Rev 39:17–43. 17364879 10.1080/03602530600952172

[8] Cavaleri M, Riva S, Valagussa A, Guanci M, Colombo L, Dowell J, Stogniew M 2005 Pharmacokinetics and excretion of dalbavancin in the rat. J Antimicrob Chemother 55(Suppl 2):ii31–ii35. 15750035 10.1093/jac/dki006

[9] Ong V, Flanagan S, Fang E, Dreskin HJ, Locke JB, Bartizal K, Prokocimer P 2014 Absorption, distribution, metabolism, and excretion of the novel antibacterial prodrug tedizolid phosphate. Drug Metab Dispos 42:1275–1284. doi:10.1124/dmd.113.056697. 24875463

[10] Shaw JP, Cheong J, Goldberg MR, Kitt MM 2010 Mass balance and pharmacokinetics of [14C]telavancin following intravenous administration to healthy male volunteers. Antimicrob Agents Chemother 54:3365–3371. doi:10.1128/AAC.01750-09. 20516282 PMC2916323

[11] Slatter JG, Adams LA, Bush EC, Chiba K, Daley-Yates PT, Feenstra KL, Koike S, Ozawa N, Peng GW, Sams JP, Schuette MR, Yamazaki S 2002 Pharmacokinetics, toxicokinetics, distribution, metabolism and excretion of linezolid in mouse, rat and dog. Xenobiotica 32:907–924. doi:10.1080/00498250210158249. 12419019

[12] Slatter JG, Sams JP, Easter JA, Fate GD, Chiba K, Johnson MG, Courtney M, Koets MD, Norris LR, Jones BW 2003 Assessment of radioactive residues arising from radiolabel instability in a multiple dose tissue distribution study in rats. Biol Pharm Bull 26:573–578. doi:10.1248/bpb.26.573. 12736492

[13] Tombs NL 1999 Tissue distribution of GAR-936, a broad-spectrum antibiotic, in male rats. Abstr 39th Intersci Conf Antimicrob Agents Chemother, abstr 413.

[14] Tanaka M, Ono C, Yamada M 2004 Absorption, distribution and excretion of 14C-levofloxacin after single oral administration in albino and pigmented rats: binding characteristics of levofloxacin-related radioactivity to melanin in vivo. J Pharm Pharmacol 56:463–469. doi:10.1211/0022357023141. 15099441

[15] Sugano K, Kataoka M, Mathews CC, Yamashita S 2010 Prediction of food effect by bile micelles on oral drug absorption considering free fraction in intestinal fluid. Eur J Pharm Sci 40:118–124. doi:10.1016/j.ejps.2010.03.011. 20307655

[16] Tanaka SK, Villano S, Tzanis E 2016 Single and multiple dose pharmacokinetics and tolerability of intravenous omadacycline in healthy volunteers. Abstr 26th Eur Congr Clin Microbiol Infect Dis, abstr P1319.

[17] Pena A, Carmona A, Barbosa A, Lino C, Silveira I, Castillo B 1998 Determination of tetracycline and its major degradation products by liquid chromatography with fluorescence detection. J Pharm Biomed Anal 18:839–845. doi:10.1016/S0731-7085(98)00268-4. 9919986

[18] Hoffmann M, DeMaio W, Jordan RA, Talaat R, Harper D, Speth J, Scatina J 2007 Metabolism, excretion, and pharmacokinetics of [14C]tigecycline, a first-in-class glycylcycline antibiotic, after intravenous infusion to healthy male subjects. Drug Metab Dispos 35:1543–1553. doi:10.1124/dmd.107.015735. 17537869

[19] Solon EG, Dowell JA, Lee J, King SP, Damle BD 2007 Distribution of radioactivity in bone and related structures following administration of [14C]dalbavancin to New Zealand White rabbits. Antimicrob Agents Chemother 51:3008–3010. doi:10.1128/AAC.00020-07. 17548492 PMC1932505

[20] Lawrence L, Longcor J, Li D, Reeve M, Hoover R, McEwen AB, Ford G, Wood SG 2012 Metabolism and mass balance of [14C]-delafloxacin in healthy human volunteers following intravenous administration. Abstr 52nd Intersci Conf Antimicrob Agents Chemother, abstr A-1956.

[21] Pereira DE, Degenhardt T, Fernandes P 2010 Comparison of CEM-101 metabolism in mice, rats, monkeys and humans. Abstr 50th Intersci Conf Antimicrob Agents Chemother, abstr A-687.

